# Effects of genetic variation in H3K79 methylation regulatory genes on clinical blood pressure and blood pressure response to hydrochlorothiazide

**DOI:** 10.1186/1479-5876-10-56

**Published:** 2012-03-22

**Authors:** Julio D Duarte, Issam Zineh, Ben Burkley, Yan Gong, Taimour Y Langaee, Stephen T Turner, Arlene B Chapman, Eric Boerwinkle, John G Gums, Rhonda M Cooper-DeHoff, Amber L Beitelshees, Kent R Bailey, Roger B Fillingim, Bruce C Kone, Julie A Johnson

**Affiliations:** 1 Center for Pharmacogenomics and Department of Pharmacotherapy and Translational Research, University of Florida, Gainesville, FL 32610, USA; 2 Department of Pharmacy Practice, University of Illinois at Chicago, Chicago, IL 60612, USA; 3 Office of Clinical Pharmacology, Office of Translational Sciences - CDER, U.S. Food and Drug Administration, Silver Spring, MD 20993, USA; 4 Division of Nephrology and Hypertension, Mayo Clinic, Rochester, MN 55905, USA; 5 Renal Division, Department of Medicine, Emory University, Atlanta, GA 30322, USA; 6 Human Genetics Center and Institute of Molecular Medicine, University of Texas Health Science Center, Houston, TX 77030, USA; 7 Division of Endocrinology, Diabetes and Nutrition, Department of Medicine, University of Maryland, Baltimore, MD 21201, USA; 8 Division of Biomedical Statistics and Informatics, Mayo Clinic, Rochester, MN 55905, USA; 9 Department of Community Dentistry and Behavioral Science, University of Florida, Gainesville, FL 32610, USA; 10 Division of Nephrology, Hypertension & Renal Transplantation, University of Florida, Gainesville, FL 32610, USA; 11 Division of Renal Diseases and Hypertension, University of Texas Health Science Center, Houston, TX 77030, USA

**Keywords:** Pharmacogenomics, Pharmacogenetics, hydrochlorothiazide, hypertension, blood pressure, *DOT1L*, *SIRT1*, *MLLT3*, *SGK1*, histone methylation

## Abstract

**Background:**

Nearly one-third of the United States adult population suffers from hypertension. Hydrochlorothiazide (HCTZ), one of the most commonly used medications to treat hypertension, has variable efficacy. The renal epithelial sodium channel (ENaC) provides a mechanism for fine-tuning sodium excretion, and is a major regulator of blood pressure homeostasis. *DOT1L, MLLT3, SIRT1*, and *SGK1 *encode genes in a pathway that controls methylation of the histone H3 globular domain at lysine 79 (H3K79), thereby modulating expression of the ENaCα subunit. This study aimed to determine the role of variation in these regulatory genes on blood pressure response to HCTZ, and secondarily, untreated blood pressure.

**Methods:**

We investigated associations between genetic variations in this candidate pathway and HCTZ blood pressure response in two separate hypertensive cohorts (clinicaltrials.gov NCT00246519 and NCT00005520). In a secondary, exploratory analysis, we measured associations between these same genetic variations and untreated blood pressure. Associations were measured by linear regression, with only associations with *P *≤ 0.01 in one cohort and replication by *P *≤ 0.05 in the other cohort considered significant.

**Results:**

In one cohort, a polymorphism in *DOT1L *(rs2269879) was strongly associated with greater systolic (*P *= 0.0002) and diastolic (*P *= 0.0016) blood pressure response to hydrochlorothiazide in Caucasians. However, this association was not replicated in the other cohort. When untreated blood pressure levels were analyzed, we found directionally similar associations between a polymorphism in *MLLT3 *(rs12350051) and greater untreated systolic (*P *< 0.01 in both cohorts) and diastolic (*P *< 0.05 in both cohorts) blood pressure levels in both cohorts. However, when further replication was attempted in a third hypertensive cohort and in smaller, normotensive samples, significant associations were not observed.

**Conclusions:**

Our data suggest polymorphisms in *DOT1L, MLLT3, SIRT1*, and *SGK1 *are not likely associated with blood pressure response to HCTZ. However, a possibility exists that rs2269879 in *DOT1L *could be associated with HCTZ response in Caucasians. Additionally, exploratory analyses suggest rs12350051 in *MLLT3 *may be associated with untreated blood pressure in African-Americans. Replication efforts are needed to verify roles for these polymorphisms in human blood pressure regulation.

## Background

Hydrochlorothiazide (HCTZ) is one of the most commonly prescribed antihypertensive drug in the US, with approximately 118 million prescriptions dispensed in 2010, either alone or combined with another antihypertensive [1,2]. HCTZ and other thiazide diuretics are recommended by current hypertension treatment guidelines in the United States as first-line treatment for most patients with uncomplicated essential hypertension, and are strongly recommended for all patients requiring two or more antihypertensives for blood pressure control [3].

Patient response to thiazides varies widely, with differential responses between and within races [4]. Because of this, clinicians have difficulty predicting which patients will achieve good blood pressure response with thiazide treatment. Pharmacogenetic studies can not only help explain this variability in drug response, but can also provide further information on the mechanistic basis of thiazides.

Thiazides achieve their initial diuretic action by preventing renal sodium reabsorption via inhibition of the Na^+^/Cl^- ^cotransporter (NCC) in the distal convoluted tubule [5-7]. However, the mechanism by which thiazides chronically lower blood pressure remains poorly understood. Also involved in sodium reabsorption is the distally-located epithelial sodium channel (ENaC). Although ENaC contributes to the reabsorption of approximately 5% of total filtered sodium load, it provides a fine-tuning mechanism for sodium, body fluid volume, and, ultimately, blood pressure homeostasis [8]. Because ENaC is distal to NCC in the nephron, inhibition of NCC, such as occurs with thiazide therapy, results in altered ion concentrations in the tubular lumen, particularly increased sodium concentrations at ENaC-expressed regions. Consequently, the clinical effect of variations in ENaC expression could be magnified in thiazide-treated patients. In fact, evidence already exists showing association between variation in *NEDD4L*, a gene involved in ENaC regulation, and blood pressure response to diuretics [9]. In addition, pharmacogenetic research has previously implicated ENaC in thiazide response, as polymorphisms in *SCNN1G *(which encodes the ENaCγ subunit) have been associated with HCTZ response [10,11]. In addition, ENaC is expressed in the vascular smooth muscle and may also play some role in regulating vascular resistance [12].

An epigenetic pathway was recently discovered that regulates ENaCα expression in the kidney by methylation of histone protein H3 at lysine 79 (H3K79) [13-15]. At the center of this pathway is a complex including the methyltransferase Disruptor of telomeric silencing 1 (Dot1) and DNA-binding protein ALL1 fused gene from chromosome 9 (Af9) [15]. Af9 (in humans, encoded by *MLLT3*) binds to the ENaCα promoter and localizes Dot1 for di- and tri-methylation at H3 Lys79, which is associated with ENaCα gene repression [16]. This repression can be prevented by Serum/glucocorticoid-induced kinase (encoded by *SGK1*), which disrupts the assembly of the Af9/Dot1 complex [14]. Evidence indicates that the deacetylase Sirtuin-1 (encoded by *SIRT1*) can also form a complex with Dot1 to decrease ENaCα expression. However, the mechanism for this interaction is still unclear [17].

We hypothesized that genetic variation in this epigenetic regulatory pathway plays a role in the antihypertensive effects of thiazides, through its regulation of ENaC. Secondarily, we hypothesized that variation in this pathway affects human blood pressure homeostasis. To test the first hypothesis, we evaluated whether polymorphisms in *DOT1L, MLLT3, SIRT1*, and *SGK1 *affect clinical blood pressure response to HCTZ in well-defined clinical cohorts. To test the second, we assessed associations of these polymorphisms with untreated clinical blood pressures as an exploratory analysis.

## Methods

### Participants

Study participants arose from the Pharmacogenomic Evaluation of Antihypertensive Responses (PEAR; clinicaltrials.gov #NCT00246519) and the Genetic Epidemiology of Responses to Antihypertensives (GERA; clinicaltrials.gov #NCT00005520). Both studies were approved by the institutional review boards at each center where they were conducted, and all subjects provided informed, written consent before being screened for enrollment.

PEAR was a multi-center clinical trial examining the role of genetic variability on blood pressure response to HCTZ and/or atenolol [18]. Men and women of any race between the ages of 17 and 65 with essential hypertension (clinic diastolic blood pressure ≥ 90 mmHg, ≤ 110 mmHg) were recruited to participate. After a four-week antihypertensive washout, included participants were randomized to receive either HCTZ 12.5 mg daily or atenolol 50 mg daily, with most receiving dose escalations to 25 mg and 100 mg, respectively for blood pressure greater than 120/70 mmHg. To assure a four-week washout sufficiently erased blood pressure effects of any previous antihypertensive treatments, we confirmed that post-washout blood pressure levels in previously-treated participants were nearly identical to those who had never received antihypertensive medication. After nine weeks, blood pressure response was assessed and for blood pressure greater than 120/70 mmHg, the other study drug was added with another dose titration and response assessment after six to nine weeks. Participants were not given sodium restrictions, but were counseled to maintain consistent dietary intakes. The primary response phenotype was home blood pressure, which participants were required to take in triplicate upon rising and before retiring at least five of seven days prior to their blood pressure assessment visit. The coefficients of variance were approximately 7% for both systolic and diastolic measurements. The first 297 self-reported Caucasians and African-Americans to complete HCTZ monotherapy (PEAR HCTZ) are included in this report. In these participants randomized to HCTZ, greater than 90% received dose increases to 25 mg. For Caucasian and African-Americans randomized to atenolol (PEAR ATEN), 374 participants were included for untreated blood pressure analyses.

GERA was a two-center clinical trial designed to determine whether polymorphisms in renin-angiotensin-aldosterone system genes were predictive of the blood pressure response to HCTZ [19]. Briefly, participants were self-reported non-Hispanic Caucasians and African-Americans between the ages of 30 and 59 who had blood pressure greater than 140/90 mmHg or a previous diagnosis of essential hypertension and current antihypertensive prescription. Previously treated hypertensives had all antihypertensive drugs discontinued for four weeks, then were assessed for blood pressure at baseline. If diastolic blood pressure remained between 90 and 110 mmHg, participants were included in the study and treated with HCTZ 25 mg daily for four weeks. Participants were counseled to stabilize sodium intake at about 1 mmol/kg/day beginning at washout and continuing throughout the study period. All blood pressure measurements were made in a clinic setting.

Two normotensive samples were also used in an attempt to replicate untreated blood pressure associations found in GERA and PEAR African-Americans. The first sample drawn from was the Ethnic Pain Sensitivity trial, a single center study designed to examine ethnic differences in pain sensitivity [20]. 206 healthy men and women between the ages of 18-53, representing multiple ethnic groups, were enrolled at the University of Florida. Mean resting systolic and diastolic blood pressure, mean arterial pressure, and mean resting heart rate were measured in each participant. For replication purposes, 88 available African-American participants were included in analyses. The second sample was a hypertension database from the University of Florida that enrolled 730 participants, both hypertensive and normotensive. Normotensive participants (systolic blood below 140 mm Hg and diastolic blood pressure below 90 mm Hg) were never diagnosed with high blood pressure and had no parents, siblings, or children with high blood pressure diagnosed before age 65. For replication purposes, 121 available African-American normotensives were included in analyses.

### Selection of polymorphisms and determination of genotypes

A tagSNP approach was used to assure maximum coverage of common SNPs in each candidate gene region. A tagSNP selection tool using the Multipop-TagSelect algorithm [21] provided online by the Genome Variation Server http://gvs.gs.washington.edu/GVS/ was queried for the genetic regions of *DOT1L, MLLT3, SIRT1*, and *SGK1 *in the HapMap YRI (African Ancestry) and CEPH (European Ancestry) populations. SNPs with a minor allele frequency less than 5% were excluded. The Genome Variation Server provided a comprehensive list of 144 SNPs meeting these criteria which tagged all four gene regions.

To more precisely investigate the strongest candidate SNPs, putative functional SNPs (pfSNPs) were added to the lists generated by the Genome Variation Server. These were computed *in silico *by two separate programs, Pupasuite [22] and FastSNP [23]. From this combined list of pfSNPs, those with a minor allele frequency greater than 0.05 for either African or European ancestry were added to the already established tagSNP list. The resulting final list contained 180 SNPs to genotype (Additional file 1; Table S1).

Genotypes in HCTZ-treated GERA and PEAR participants were determined using a custom GoldenGate Assay for the BeadXpress Reader System (Illumina Inc., San Diego, CA). Genotyping was carried out according to the manufacturer's protocol. Raw data conversion and quality control were completed in GenomeStudio software (Illumina Inc., San Diego, CA). Samples were excluded if their genotype call rate was below 90%. Individual SNPs were excluded from analysis if they were monomorphic in our cohorts, their call frequencies were below 75%, or their GenTrain scores were less than 0.3.

For untreated blood pressure replication analyses, genotypes were determined using Taqman SNP Genotyping Assays and the Taqman 7900HT Real Time PCR System (Applied Biosystems, Foster City, CA) according to the manufacturer's protocol.

### Statistical methods

Associations between genotype and blood pressure responses to HCTZ were tested by linear regression after adjustment for covariates, including gender, age, and untreated blood pressure. Associations between untreated systolic blood pressure and diastolic blood pressure were tested in the same manner as described for HCTZ response, except covariate adjustments included only gender and age. Statistical analyses were completed in JMP Genomics 4 and SAS 9.2 (SAS Institute, Cary, NC).

Because of the large number of SNPs tested, adjustments for multiple comparisons were necessary to avoid false positives. A Bonferroni correction, assuming 180 independent tests, would require a *P *≤ 0.00028 for significance (0.05/180 = 0.00028). However, the SNPs analyzed are strong biological candidates, and do not represent independent tests due to linkage disequilibrium. Additionally, replication of findings is essential in genetic association studies. In our analyses, SNPs were considered significant if they associated with HCTZ blood pressure response in either GERA or PEAR with *P *≤ 0.01, replicated with *P *≤ 0.05 in the other study group, and had matching directions of effect. With this replication strategy, the overall *P*-value threshold for significance is 0.01 × 0.05 × 0.5 (for matching direction of effect) = 0.00025, which therefore meets the Bonferroni criterion, as similarly justified previously [24].

As a quality control procedure, Hardy-Weinberg equilibrium was tested via χ^2 ^analysis separately by race and study group. Those SNPs with Hardy-Weinberg equilibrium *P*-values less than a Bonferroni-corrected 0.00028 were flagged and analyzed under suspicion of genotyping error.

## Results

### Study cohorts

Baseline characteristics were similar among both study cohorts (Table 1). Both normotensive replication cohorts varied from the study cohorts in racial make-up and untreated blood pressure (by design), and also varied slightly from all other groups in age and BMI. In PEAR, HCTZ treatment decreased blood pressure by approximately 12/7 mmHg in African Americans and 8/4 mmHg in Caucasians. In GERA, HCTZ treatment decreased blood pressure by approximately 18/9 mmHg in African Americans and 11/6 mmHg in Caucasians.

**Table 1:** Baseline demographics of GERA and PEAR clinical cohorts

	GERA	PEAR HCTZ	PEAR ATEN	HTNDB AA Normotensives	EPS AA Population
N	583	297	374	121	88
Age (y)	48.2 ± 6.7	50.3 ± 8.9	48.6 ± 9.2	45.1 ± 7.1	22.7 ± 5.1
Sex (% female)	47.2	51.2	57.7	52.9	51.1
Race (%)					
Caucasian	50.3	57.1	58.9	0	0
African-American	49.7	42.9	41.1	100	100
BMI (kg/m^2^)	31.3 ± 6.0	30.8 ± 5.6	31.0 ± 6.2	30.1 ± 6.8	25.2 ± 4.3
Mean Clinic BP					
Systolic (mmHg)	146.0 ± 14.4	152.6 ± 13.0	151.4 ± 12.4	118.3 ± 9.1	122.5 ± 8.9
Diastolic (mmHg)	96.1 ± 5.4	98.7 ± 6.1	98.3 ± 6.2	76.7 ± 6.3	68.4 ± 5.9
Mean Home BP					
Systolic (mmHg)	N/A	146.7 ± 11.2	145.2 ± 9.8	N/A	N/A
Diastolic (mmHg)	N/A	94.3 ± 6.2	93.5 ± 6.0	N/A	N/A

Values are listed as mean ± SD

GERA - Genetic Epidemiology of Responses to Antihypertensives trial, PEAR HCTZ - Randomized to HCTZ in the Pharmacogenomic Evaluation of Antihypertensive Responses trial, PEAR ATEN - Randomized to atenolol in the Pharmacogenomic Evaluation of Antihypertensive Responses trial, EPS AA population - African Americans from the Ethic Pain Sensitivity trial, HTNDB AA Normotensives - Normotensive African Americans from a University of Florida hypertension database, BP - blood pressure, N/A - Not Available.

Out of a total 180 SNPs genotyped with the GoldenGate assay, six SNPs were excluded for having a SNP call frequency less than 75% or a GenTrain score less than 0.3. An additional two SNPs failed these quality controls in GERA only, so were analyzed exclusively in PEAR. In GERA, 19 participant samples were excluded from analysis because of low genotype call rates (< 90%). In PEAR, one participant sample was excluded from analyses because of low call rates. Taqman genotyping of rs12350051 was completed in replication samples with approximately 7% duplication, revealing 98% concordance. Both rs2269879 and rs12350051 were in Hardy-Weinberg equilibrium in all groups analyzed.

### Association of candidate gene variation with hydrochlorothiazide response

**Figure 1:**
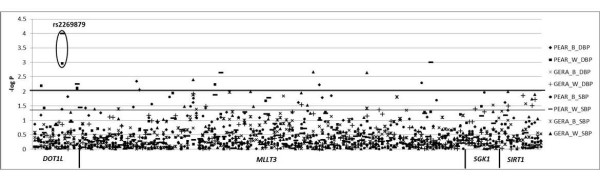
**Association of candidate SNPs with blood pressure response to HCTZ in GERA and PEAR HCTZ cohorts**. The lower line represents *P *= 0.05, while the higher line represents *P *= 0.01. B - African American, W - Caucasian, BL - Untreated, SBP - systolic blood pressure response, DBP - diastolic blood pressure response.

**Figure 2:**
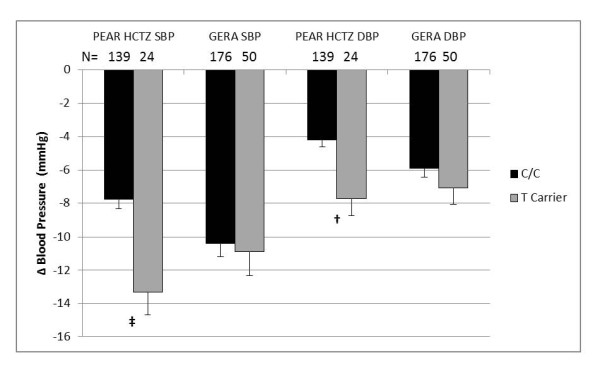
**Blood pressure response to HCTZ by rs2269879 genotype in GERA and PEAR in Caucasian samples**. Adjusted for age, gender, and untreated blood pressure. Error bars indicate standard error. SBP - Systolic blood pressure; DBP - diastolic blood pressure; ‡ - *P *≤ 0.001; † - *P *≤ 0.01.

A Manhattan plot of -log *P*-values for genetic associations with blood pressure response in GERA and PEAR HCTZ (Figure 1) indicate no SNP associations were replicated according to predetermined criteria for significance. The *DOT1L *SNP rs2269879 came the closest to our criteria for a significant association. The variant T allele at rs2269879 was associated with a 5.5 mmHg greater mean systolic (*P *= 0.0002) and a 3.5 mmHg greater mean diastolic (*P *= 0.0016) response to HCTZ in PEAR HCTZ Caucasians (Figure 2), while no association was found in PEAR HCTZ African-Americans. Blood pressure response associations in GERA Caucasians possessed the same direction of effect as those in PEAR HCTZ (Figure 2), but were not statistically significant (systolic *P *= 0.73, diastolic *P *= 0.29). Because GERA *P*-values did not meet our predetermined criteria for significance, this SNP was not considered a replicated association.

### Association of candidate gene variation with untreated blood pressure

**Figure 3:**
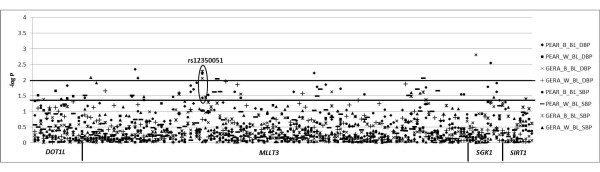
**Association of candidate SNPs with untreated blood pressure in GERA and PEAR HCTZ cohorts**. The lower line represents *P *= 0.05, while the higher line represents *P *= 0.01. B - African American, W - Caucasian, BL - Untreated, SBP - systolic blood pressure, DBP - diastolic blood pressure.

**Figure 4:**
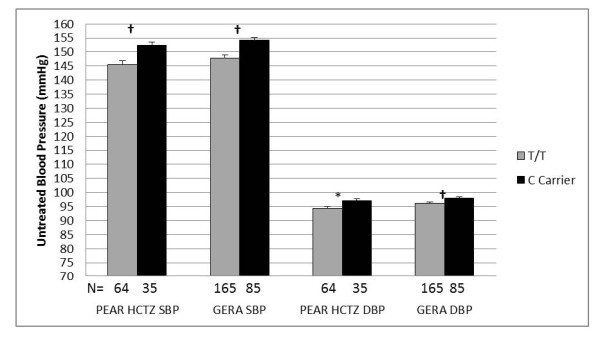
**Untreated blood pressure by rs12350051 genotype in GERA and PEAR HCTZ African-American samples**. Adjusted for age and gender. Error bars indicate standard error. SBP - Systolic blood pressure; DBP - diastolic blood pressure; † - *P *(trend) ≤ 0.01; * - *P *(trend) ≤ 0.05.

In a pre-defined secondary analysis, we observed associations between baseline, untreated blood pressure and rs12350051 (in *MLLT3*) genotype in both PEAR HCTZ (systolic *P *= 0.005, diastolic *P *= 0.049) and GERA African-Americans (systolic *P *= 0.001, diastolic *P *= 0.010) at the pre-defined thresholds for association and replication (Figure 3). African-Americans with the variant C allele at rs12350051 had mean untreated systolic blood pressures 6.4 mmHg higher in PEAR HCTZ and 6.9 mmHg higher in GERA than those with the T/T genotype (Figure 4). Mean untreated diastolic blood pressures were 2.8 mmHg higher in PEAR HCTZ and 1.8 mmHg higher in GERA (Figure 4). No association was observed in Caucasians.

**Figure 5:**
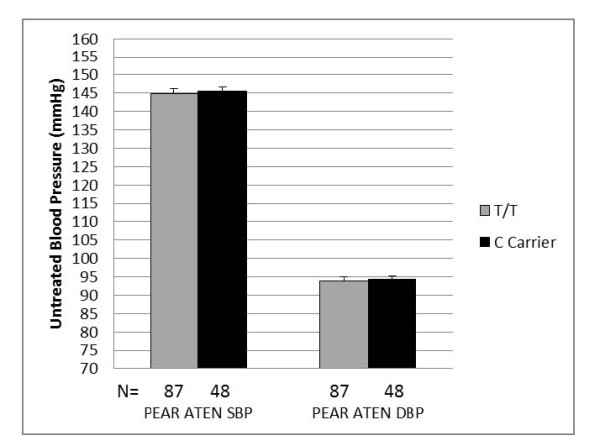
**Untreated blood pressure by rs12350051 genotype in PEAR ATEN African-American sample**. Adjusted for age and gender. Error bars indicate standard error. SBP - Systolic blood pressure; DBP - diastolic blood pressure.

Since this discovered association was with untreated blood pressure, baseline data could be used from African-Americans randomized to atenolol in PEAR ATEN as a further replication. While the untreated blood pressures of PEAR ATEN African-Americans followed a similar trend to GERA and PEAR HCTZ African-Americans, the means between genotype groups differed by less than 1 mmHg for both systolic and diastolic blood pressures and the difference was not statistically significant (Figure 5). Further association testing in the African-American normotensive replication samples revealed no associations with untreated blood pressure (data not shown).

## Discussion

We investigated associations in a candidate pathway for HCTZ blood pressure response, which controls expression of ENaC. Our study showed no association between common variation in *DOT1L, MLLT3, SIRT1*, or *SGK1 *and blood pressure responses to HCTZ that met our pre-defined criteria for significance. In an exploratory analysis, we also evaluated the role of SNPs in this pathway on untreated blood pressure and identified rs12350051 in *MLLT3 *as being associated with baseline blood pressure in both GERA and PEAR African-Americans. However, in another PEAR and two normotensive cohorts, this association did not replicate.

The strongest pharmacogenetic association with HCTZ response was with rs2269879 in DOT1L, and was only observed in PEAR Caucasians. Results with systolic and diastolic response in GERA were directionally consistent, but nonsignificant. Located in intron 7, rs2269879 was chosen for genotyping as a tagSNP. Upon review in the HapMap CEPH population, the SNP was found to be in perfect linkage with rs8113528 (r^2 ^= 1.0), in intron 3. FastSNP showed the variant A allele at rs8113528 creates a possible binding site for p300, transcriptional co-activator that functions as a histone acetyltransferase. Additionally, UCSC Genome Browser http://genome.ucsc.edu/ indicates rs8113528 exists in an area surrounded by moderate histone acetylation.

Because the association only met the significance threshold in PEAR, and did not replicate in GERA, we cannot rule out that this is a chance finding. One reason for a lack of replication in GERA may be that the effect of this SNP can only be detected using home blood pressure. PEAR was the only study with the home blood pressure phenotype available. We decided *a priori *to use it as the response phenotype in PEAR because home blood pressure is a more accurate phenotype, as home blood pressure predicts cardiovascular risk better than office blood pressure [25,26]. In addition, we previously found ambulatory blood pressure measurement, another potentially better predictor of cardiovascular risk, correlated with home blood pressure more than with office blood pressure in a subset of PEAR participants [27]. PEAR home blood pressure entries were averages of multiple measurements spanning at least five days, thus they likely give a better estimate of participants' actual blood pressures. Home blood pressure is also a more precise phenotype, as evidenced by the smaller standard deviations in home systolic blood pressure measurements we observed in PEAR compared with office measurements (Table 1 systolic: *P *< 0.001, diastolic: *P *= 0.689). Office measurements, the only blood pressure phenotypes available in GERA, may not possess high enough fidelity to detect this association with rs2269879. Supporting this theory, we observed similar, but much weaker associations with office blood pressure response in PEAR. Perhaps if another large hypertensive cohort, prospectively treated with HCTZ, becomes obtainable for analysis of home blood pressure responses, the association we found could be tested again for replication.

The lack of association we found with HCTZ response suggests that genotyping polymorphisms in this pathway would likely not help predict patient response to thiazide diuretics. The likelihood that common SNP associations were missed with *DOT1L, MLLT3, SIRT1, SGK1 *and blood pressure associations is low. TagSNPs within 5000 bases of each candidate gene were selected to try to detect any possible *cis*-regulatory regions. Great effort was spent on identifying pfSNPs *in silico *for each candidate gene, which were not required to be in the pre-defined gene region for tagSNP development. However, only SNPs with a minor allele frequency of 0.05 were considered for genotyping, so our study cannot rule out very rare SNPs in the candidate genes with large effect sizes affecting blood pressure response. Additionally, our data do not rule out whether or not this pathway plays any role thiazide response. If HCTZ did have some small effect on H3K79 methylation, redundancy in ENaCα regulation [28] could conceivably overcome the changes in H3K79 methylation and leave behind no measurable change in patient blood pressure response.

Little is known about the effect of this histone H3K79 methylation pathway on blood pressure regulation in humans, so exploratory analyses testing associations in untreated blood pressure phenotypes could also provide valuable information. The SNP that associated and replicated with untreated blood pressure was rs12350051 in *MLLT3*. It was chosen as a tagSNP, and is located in intron 2, with no linkage to any known functional SNPs. *In silico*, rs12350051 was not observed in any known miRNA sequences, transcription factor binding sites, exonic splice sites, splice enhancer, or silencer sequences. Because the same blood pressure association was not seen in Caucasians, one possibility could be that this SNP is in high linkage disequilibrium with an undiscovered functional polymorphism in African-Americans.

One would expect using a patient population with a wide range of blood pressures to be the best method to detect genetic associations with untreated blood pressure. So the fact that untreated blood pressure associations were seen in PEAR and GERA is somewhat surprising, as these studies enrolled hypertensives spanning a relatively small blood pressure range. This was one of the reasons we attempted to replicate these findings in normotensive blood pressure ranges not represented in PEAR and GERA. However, the normotensive groups also had a narrow blood pressure range. The fact that no replication was observed in normotensives could be because of this narrow blood pressure range, the fact that they were younger, the differences in study protocols, leading to differences in blood pressure measurement precision, or the sample sizes were too small and lacked the power to detect the effect we observed in the larger hypertensive cohorts. Another possibility is that perhaps the effect of this SNP is easier to detect or only exerts an effect with higher blood pressures.

Our findings are not the first to detect associations between this H3K79 methylation pathway and blood pressure regulation. *Dot1 *conditional knockout mice were shown to exhibit salt sensitive hypertension [29]. Conversely, mice null for *Af17*, which has been shown to compete with Af9 for Dot1 binding at the ENaCa promoter, [30] exhibit renal salt wasting and hypotension with diminished renal H3K79 methylation and renal ENaCα gene expression [31]. Also, Sirtuin-1 deacetylation of endothelial nitric oxide synthase [32] has been suggested as a possible mechanism for the blood pressure reduction seen during caloric restriction, a well-known inducer of Sirtuin-1 [33].

## Conclusions

In conclusion, associations likely do not exist between common variation in *MLLT3, SIRT1*, or *SGK1 *and blood pressure responses to HCTZ in hypertensives. One SNP in *DOT1L *(rs2269879) could play a role in HCTZ response, but requires further investigation to replicate the association found in PEAR. Additionally, rs12350051 in *MLLT3 *was associated with untreated blood pressure in African-American hypertensive individuals. Because this was an exploratory analysis, and the association was not replicated in smaller normotensive samples, questions remain as to whether this polymorphism is involved in the blood pressure regulation of normotensives, and the mechanism by which rs12350051 exerts an effect on blood pressure. Further study in clinical populations with broader blood pressure ranges would help answer these questions.

## Abbreviations

ATEN: atenolol; HCTZ: hydrochlorothiazide; H3K79: histone H3 lysine 79; NCC: sodium - chloride cotransporter; ENaC: epithelial sodium channel; ENaCα: epithelial sodium channel α subunit; SNP: single nucleotide polymorphism; pfSNP: putative functional SNP; GERA: Genetic Epidemiology of Responses to Antihypertensives; PEAR: Pharmacogenomic Evaluation of Antihypertensive Responses.

## Competing interests

The authors declare that they have no competing interests.

## Authors' contributions

JDD participated in the study design, participated in genotyping, performed statistical analyses and drafted the manuscript. IZ participated in the design of the study and helped to draft the manuscript. BB participated in sample processing and genotyping and helped to draft the manuscript. KRB assisted with statistical analysis, STT and ALB participated in study coordination and helped to draft the manuscript. YG, TYL, ABC, EB, JGG, RMC, RBF participated in study coordination. BCK conceived the study, participated in its design, and helped to draft the manuscript. JAJ conceived the study, participated in its design, and helped to draft the manuscript. All authors read and approved the final manuscript.

## Supplementary Material

Additional file 1 **Supplementary Table 1**. SNPs genotyped in *SGK1, DOT1L, SIRT1*, and *MLLT3 *gene regions. Click here for file
