# Supplementary material for: Effects of genetic variation in H3K79 methylation regulatory genes on clinical blood pressure and blood pressure response to hydrochlorothiazide
Source: J Transl Med. 2012 Mar 22;10:56. doi: 10.1186/1479-5876-10-56 (PMC3320544; doi:10.1186/1479-5876-10-56)
Supplement: Additional file 1 — Supplementary Table 1. SNPs genotyped in SGK1, DOT1L, SIRT1, and MLLT3 gene regions. [file 1479-5876-10-56-S1.DOC]

Supplementary Table 1. SNPs genotyped in *SGK1*, *DOT1L*, *SIRT1*, and *MLLT3* gene regions

| ***SGK1*** | rs2302061 | rs3740051 | rs10811350 | rs2383138 | rs7039016 |
| --- | --- | --- | --- | --- | --- |
| rs9402571 | rs8113174 | rs10997866 | rs10811352 | rs2519454 | rs7041793 |
| rs7752937 | rs8113528 | rs12778366 | rs10811358 | rs2775265 | rs7042746 |
| rs1763527 | rs7256735 | rs752578 | rs10811362 | rs2780839 | rs7046522 |
| rs1057293 | rs12459350 | rs35671182 | rs10811372 | rs2780840 | rs7047471 |
| rs7755303 | rs12462556 | ***MLLT3*** | rs10811376 | rs2780841 | rs7874260 |
| rs6911375 | rs12611107 | rs10964538 | rs10964542 | rs3764797 | rs7874909 |
| rs3813344 | rs12981806 | rs4977421 | rs10964545 | rs3780826 | rs10964539 |
| rs2758152 | rs12983678 | rs10964597 | rs10964547 | rs3780829 | rs16938091 |
| rs2758151 | rs1558118 | rs6475454 | rs10964552 | rs3780830 | rs3824576 |
| rs2758150 | rs886449 | rs10121168 | rs10964555 | rs6475462 | rs6475455 |
| rs12663728 | ***SIRT1*** | rs4977256 | rs10964562 | rs668703 | rs10964600 |
| rs1743966 | rs34639502 | rs7043548 | rs10964566 | rs7018627 | rs10964605 |
| ***DOT1L*** | rs1966188 | rs1016962 | rs10964593 | rs7020320 | rs10964620 |
| rs7251881 | rs2234975 | rs10738563 | rs16938109 | rs7020494 | rs10964636 |
| rs1003531 | rs35671182 | rs10757132 | rs17177890 | rs7020850 | rs1111766 |
| rs2074552 | rs3758391 | rs10811359 | rs1929170 | rs7023694 | rs11999881 |
| rs2240130 | rs7069102 | rs10757140 | rs2073853 | rs7025895 | rs12003167 |
| rs2269879 | rs7096385 | rs10811346 | rs2188229 | rs7029191 | rs12004010 |
| rs2269882 | rs36107781 | rs10811348 | rs2188230 | rs7029476 | rs12004720 |
| rs2286329 | rs35706870 | rs12685798 | rs2301550 | rs7036342 | rs12005512 |

| ***MLLT3*** | rs12380857 | rs1633699 | rs3904577 | rs623828 | rs1411727 |
| --- | --- | --- | --- | --- | --- |
| rs12006253 | rs12554291 | rs16925395 | rs3934325 | rs6475431 | rs7866601 |
| rs12335409 | rs12682679 | rs16938035 | rs4246840 | rs6475442 | rs7866741 |
| rs12337265 | rs12683597 | rs16938039 | rs4448374 | rs6475444 | rs7868378 |
| rs12342870 | rs1291635 | rs16938042 | rs4977257 | rs6475448 | rs7870252 |
| rs12344417 | rs13289029 | rs16938054 | rs4977425 | rs6475451 | rs7873127 |
| rs12346032 | rs13299141 | rs16938062 | rs4977427 | rs734477 | rs7874260 |
| rs12350051 | rs1360378 | rs16938065 | rs4977430 | rs734772 | rs7037941 |
| rs12377027 | rs1537069 | rs3892039 | rs4621895 | rs7854241 | rs12006034 |
| rs12379192 | rs1548375 | rs3897271 | rs5021140 | rs7858673 |  |
